# Epidemiological Study of Autism Spectrum Disorders in Greece for 2021: Nationwide Prevalence in 2–17-Year-Old Children and Regional Disparities

**DOI:** 10.3390/jcm12072510

**Published:** 2023-03-27

**Authors:** Raissa Kouznetsov, Panagiotis Angelopoulos, Spyridon Moulinos, Ioannis Dimakos, Philippos Gourzis, Eleni Jelastopulu

**Affiliations:** 1 Department of Public Health, School of Medicine, University of Patras, 26504 Patras, Greece; 2 Independent Researcher, New York, NY 10010, USA; 3 Department of Digital Media and Communication, Ionian University, 49100 Kerkira, Greece; 4 Department of Educational Sciences and Social Work, University of Patras, 26504 Patras, Greece; 5 Department of Psychiatry, School of Medicine, University of Patras, 26504 Patras, Greece

**Keywords:** autism spectrum disorder, autism, prevalence, Greece, epidemiology, public health, data

## Abstract

This study estimated the crude prevalence of autism spectrum disorders (ASD) in Greece in 2021. A retrospective study was conducted for 2021 using data provided for research purposes for the first time by the Greek National Health Service Organization for Healthcare Services Provision (EOPYY) related to the ICD-10 diagnosis codes F84.0–F84.9 (ASD). Treatments were categorized by gender, age, and location. Statistical analysis was performed using the open-source software R. In total, 15,706 children aged 2–17 years were registered with ASD: 12,380 boys and 3326 girls. In total, 6,117,910 therapies were prescribed: 4,844,173 for boys and 1,273,737 for girls. Boys are estimated to be diagnosed 3.5 times more often than girls. On average, approximately 390 treatments are prescribed per person per year for both sexes. The annual prevalence is estimated at 0.94%, ranging from 0.42% to 1.44% depending on geographic region. Our findings provide evidence-based data for the planning of policies regarding health, education, and employment for people with ASD. The number of children and treatments makes ASD a public health concern to support children and their families and ensure equal participation in all aspects of society.

## 1. Introduction

Autism spectrum disorders (ASD) are a key public health issue [1]. Autism has emerged as a major public health concern over the last few decades because the prevalence of ASD is continuously increasing worldwide [2]. A systematic review from November 2021 showed a median global prevalence of 100/10,000 [2]. Autism has been considered one of the most severe childhood neurodevelopmental disorders for almost eighty years, although for the first fifty years it was thought to be quite rare [3]. Nowadays, autism is recognized as a broader spectrum that affects individuals in several visible and invisible ways [4,5,6]. Individuals with ASD present difficulties in social skills and communication, as well as difficulties in interaction with other people, and they often have special and repetitive interests, stereotypical behaviors, and movement difficulties [7]. This broader spectrum includes autonomous people with limited difficulties, as well as people with severe symptoms, with several disabilities and possible intellectual disability, who need lifelong support [8]. Many studies have shown that ASD are a neurobiological disorder influenced by both genetic and environmental factors affecting the developing brain and determine the factors that correlate with a risk of developing ASD [9]. Children and adults with ASD experience multiple anxieties, such as fear of the unknown, because it is difficult to expose themselves to unpredictable environments [10]. ASD is diagnosed based on clinical criteria, as described in the International Classification of Diseases (ICD-10) of the World Health Organization (WHO) or in the Psychiatric Disorders (DSM-5) of the American Psychiatric Association [7,11].

The cause of ASD has not yet been identified [12]. Studies are ongoing to determine the possible risk factors, with the latest studies emphasizing genetic and environmental aspects [9]. References have also been made to the possible association of older parental age in relation to the occurrence of ASD [12].

Applying a public health approach to autism research [13] is critical because many studies focus on basic clinical research. Public health focuses on finding health determinants to improve individuals’ quality of life and protect vulnerable populations.

This study aimed to present epidemiological data for ASD in Greece and to estimate the prevalence of autism spectrum disorders in children aged 2–17 years for the year 2021. This is the first study conducted for this age group in Greece with data provided by the Greek National Health Service Organization for Healthcare Services Provision (EOPYY). Furthermore, differences in the ASD diagnosis and treatment prescription between genders and regions were investigated.

It is crucial to assess the data for children with ASD in order to plan the appropriate health and educational policies to support these children and their families [14]. Affected individuals need educational, medical, and social services to help them cope with the lifelong challenges of autism.

## 2. Materials and Methods

### 2.1. Study Design

This is the first study to be provided with formal analytical administrative data by an official Greek institution regarding the age, gender, and treatments for people diagnosed with ASD. It is a retrospective study for the year 2021. The data were provided in the form of .xls files; for statistical analysis, the open-source statistical program R [15] was used.

### 2.2. Data Source—Population

Diagnoses of ASD were provided by the Greek National Organization for Healthcare Services Provision (EOPYY) for each child, in addition to the prescribed treatment. EOPYY provided a hashed social security number (AMKA) for each child without revealing any personally identifiable information. The AMKA is essentially the work and insurance ID of every employee, pensioner, and dependent member of a family in Greece. In addition to the hashed AMKA, the child’s age category, gender, and region were provided with the year and the month the treatment was prescribed, the ICD-10 diagnostic code, the prescribed therapy, and the number of therapies for the given month. In total, there were 546,240 records in the provided dataset, corresponding to 15,706 individuals. To avoid double-counting individuals in different age categories, those who changed age group within the year were kept fixed throughout the analysis in their respective age group from the first time they appeared in the dataset during 2021.

### 2.3. Quality of Data

This is considered the most complete set of diagnoses and treatments available in Greece, as EOPYY introduced a digitized system in 2019 where doctors are required to prescribe treatments and record diagnoses based on AMKA for each patient. This recording system has been mandatory since 2020 and all therapies must be prescribed in the system so that treatment can be provided through each person’s national security number AMKA. EOPYY is the Greek National Health Service Organization for Healthcare Services Provision under the Ministry of Health.

In addition to the EOPYY data, population data were obtained from the census conducted by the Hellenic Statistical Authority (ELSTAT) for the study population. ELSTAT provided the population size for each age group and gender for each geographic region.

### 2.4. Statistical Analysis

To estimate the crude prevalence of ASD in the population, the number of unique individuals between the ages of 2 and 17 years who were assigned at least one ICD-10 code was divided by the total population of that age group, as recorded by the Hellenic Statistical Authority during the most recent census and was updated for 2021. To compare the prevalence between the genders, we employed the chi-squared test. The difference of prevalence between groups is expressed in terms of the risk of one group to be impacted versus another. For this reason, the difference in prevalence between genders in terms of an odds ratio was calculated and an odds ratio test was conducted to support the causative association between gender and prevalence. The second hypothesis investigated was whether there is a difference in the prescription of the number of therapies between genders. To examine this hypothesis, a *t*-test was conducted on the average number of therapies prescribed per individual per year. A two-way analysis of variance (ANOVA) was conducted to control for age and gender when estimating the difference in the average number of therapies prescribed per year.

Finally, to evaluate the hypothesis of whether there are regional differences in the prevalence of ASD between different regions, a univariate logistic regression model with the region as the predictor was employed. The reference level was set as the region of Attica, which is the largest region in Greece and where the standard of care is expected to be given. To perform the analysis, 1424 IDs were excluded that were missing a location from the EOPYY data but were included in the total number of children with ASD diagnosis.

## 3. Results

### 3.1. Prevalence of Autism Spectrum Disorder

The overall prevalence of ASD in Greece in the 2–17-year-old age group was calculated to be 0.94%, with males impacted at a much higher rate (1.44% vs. 0.41%; *p*-value < 0.001) and an odds ratio equal to 3.55 (C.I. [3.42, 3.69] *p*-value < 0.001). The total number of people with ASD for 2021 was 15,706 for those aged 2–17 years, out of which 12,380 diagnoses refer to boys and 3325 diagnoses refer to females. As shown in Table 1, approximately 1 out every 100 people is diagnosed with ASD and boys are 3.54 times more likely to be diagnosed with ASD than girls.

**Figure 1:**
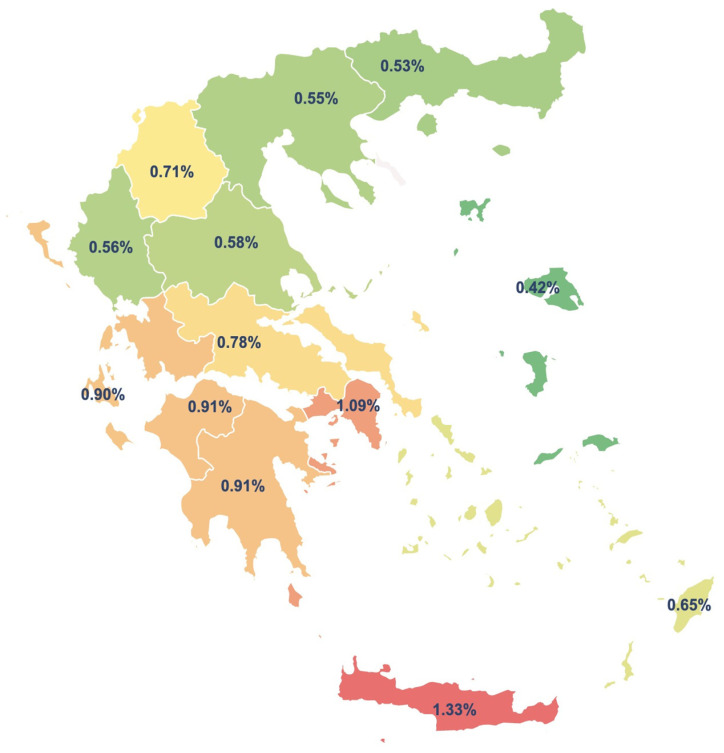
Prevalence of ASD per geographical region.

It is interesting to look at the prevalence according to geographic location. The data provided were categorized geographically according to the 13 Greek regions. As shown in Table 2 and Figure 1, the prevalence of ASD is significantly lower in northern Greece than in central and southern Greece. There is no evidence, research, or scientific literature to indicate the reasons why this occurs, although a wide range of prevalence is observed in relation to the geographic region, from 0.42% to 1.33%.

In Table 3, the output of the logistic regression model is presented. It was observed that all coefficients are significantly different than zero when executing the Wald test. The odds ratio can be interpreted as the change in risk of an individual in the different regions being diagnosed with ASD compared with the individuals in the Attica region. There are also significant differences observed across regions, with the range in the odds ratio being between 0.38 (North Aegean) and 1.21 (Crete). All regions except for Crete have an odds ratio lower than 1, indicating that the prevalence of ASD for those regions is lower than in Attica.

### 3.2. Diagnosis of Autism Spectrum Disorders for Children Aged 2–17 Years for 2021

For this analysis, ICD-10 diagnosis codes F84.0–F84.9 used for ASD were considered. According to the data, the main diagnosis codes are childhood autism (F84.0) and atypical autism (F84.1). Almost similar percentages were found for both genders, 99% for boys and 98% for girls (Table 4).

The proportion of diagnoses of ASD in relation to sex for the entire age group of 2–17 years was 78.82% for boys and 21.18% for girls. As shown in Table 5, similar percentages were observed in the different age groups with no significant variations. In Table 4 and Table 5, the total number of children with ASD exceeds the total number of 15,706; in some cases, a child may have more than one diagnosis code. However, individuals with multiple diagnostic codes for ASD are counted only once in our prevalence estimates.

### 3.3. Prescribed Treatments to Children Aged 2–17 Years with Autism Spectrum Disorders for 2021

The total number of treatments for ASD for all ages under in this study was 6,117,910. As shown in Table 6, the most prescribed treatments were speech therapy, occupational therapy for children, and special education. The total number of the three treatments was 4,646,446 therapies or 75.9% of all treatments. Thus, treatments were concentrated and not equally disbursed throughout the whole range of available therapies.

The number of treatments prescribed was 4,844,173 (79.2%) for boys and 1,273,737 for girls (20.8%). As shown in Table 7, on average, boys are prescribed eight more treatments for ASD compared with girls. Of note, the number of annual treatments exceeded the number of days in a calendar year for both sexes, meaning that some treatments were administered more than once per day. Boys are prescribed statistically significantly more treatments per year than girls, averaging 391 and 383 treatments, respectively (*p*-value 0.015; C.I. for the difference between treatments, [1.58, 15.07]).

However, to examine whether there is indeed a statistical difference in treatment prescribing between the sexes, a two-way control ANOVA was conducted for the age variable. When controlling for age, the differences between genders were no longer statistically significant (*p*-value 0.055), although age was statistically significant (*p*-value < 0.001).

Table 8 presents the prescribed therapies by age. It is obvious, that the content of the prescribed treatment differs by age. However, speech therapy is the most prescribed treatment in all age groups except of the ages 0–2 years.

## 4. Discussion

### 4.1. Key Results

In our study, the prevalence of ASD diagnosis in 2021 was estimated at 0.94%, i.e., approximately 1 in 100 children will be diagnosed with ASD (1.44% in males and 0.41% in females; male-to-female ratio 3.54:1). The odds ratio was 3.54, meaning that boys were 3.54 times more likely to be diagnosed with ASD than girls. It is important to note that girls with ASD may go undiagnosed or receive a different type of diagnosis due to their ability to camouflage their difficulties, which can delay their ASD diagnosis [16].

As can be seen from the ICD-10 codes, the diagnoses concerned 67.60% of boys and 63.96% of girls with childhood autism. Accordingly, 31.49% of boys have been diagnosed with atypical autism compared with 33.42% of girls. Of note, is the lack of use of the code F84.5 for Asperger syndrome, suggesting that children with high-functioning autism may avoid associating their diagnosis with their social security number and the health care system.

The lack of this diagnosis also reflects the difficulty in identifying this particular form of high-functioning autism by clinicians. In fact, these individuals often reach adulthood without an ASD diagnosis and are usually referred to a psychiatrist because of comorbidities, such as mood disorders, anxiety disorders, etc.

The results for the prescribed treatments are impressive. In 2021, 4,844,173 treatments were prescribed for boys and 1,273,731 treatments were prescribed for girls. It is interesting to note that the first choice for the treatment of autism spectrum disorders in Greece is speech therapy, with a percentage of 28.19%, followed by occupational therapy (27.53%) and special education, with 20.23% of the total treatments prescribed. The low percentage of family psychotherapies prescribed (0.11%) is extremely impressive. The entire family of an autistic person needs help and psychoeducation to support all members. The annual number of prescribed treatments per person is also remarkable: it was 391 for boys and 383 for girls. More specifically, for those aged 8–12 years, the annual number of treatments was 419 for boys and 422 for girls. This means that a child has to undergo two different treatments almost every day and on working days (5 days per week). This is a clear indication of the burden on the child, their family, society, and the health care system.

### 4.2. Limitations

This study analyzed administrative data provided by the Greek National Health Service Organization for Healthcare Services Provision (EOPYY), the official agency responsible for the provision of health services in Greece. Almost all Greek citizens have a social security number, under which all types of health services they receive are recorded. However, we were not able to identify any social security number with the ICD-10 diagnosis code F84.5, which indicates Asperger syndrome, in the records. This suggests that the actual prevalence of autism spectrum disorder may be even higher than we found. However, it should be noted that our study did not include children who do not have social security numbers or who are treated outside the public health system.

Another limitation of our study is related to the accuracy of ASD diagnosis by child psychiatrists, developmental pediatricians, and child neurologists. The diagnosis relies on the clinical experience and expertise of these health professionals and may be subject to underdiagnosis or misdiagnosis.

### 4.3. Interpretation

These findings are similar to the first epidemiological study on ASD performed in Greece [17], although there were differences in the child population and methodology. That study estimated the prevalence in 2019 for children aged 10 and 11 years based on administrative data from the public centers for educational and counseling support in Greece and had a population coverage of 87%. In that study, the prevalence of ASD diagnosis was calculated to be 1.15% (1.83% for males and 0.44% for females, i.e., a male-to-female ratio of 4.14:1). Additionally, a local study was conducted in Crete, where the prevalence of ASD on the island was calculated to be 0.32% in 2012 based on school record data [18]. According to our study, the prevalence of ASD in children aged 2–17 years in Crete was estimated to be 1.33% in 2021, the highest percentage of all regions in Greece. The differences between these studies are to be expected, as the age range of the population and the methodology differ significantly.

Consistent with other studies, the differences between rural areas and urban areas in Greece are thought to be likely related to differences in diagnostic procedures and the availability and accessibility of specialized services for people with ASD.

The European prevalence rate for ASD in young people aged 5–18 years was estimated to be 0.8% based on registry-based studies and 1.4% based on population [19]. These data confirm that prevalence varies worldwide, which may be due to methodological differences in case detection and data sources [20,21].

The National Autism Center in the United States of America established the National Standards Project (NSP) in 2005 to address the need for evidence-based practice guidelines for ASD [22]. Evidence-based interventions and non-pharmaceutical treatments for children with autism spectrum disorders (ASD) involve the disciplines of psychology, speech/language disorders, occupational therapy, and developmental pediatrics. The results of our study show that a person with autism and his or her family need multidisciplinary support. Effective treatments for children with ASD require an interdisciplinary approach [22]. Combinations of educational interventions, psychological/behavioral therapy, speech therapy, occupational/physical therapy, and medical treatments are required.

## 5. Conclusions

In Greece, the prevalence of autism spectrum disorder seems to be in line with the global prevalence according to the World Health Organization [23], which is almost 1 in 100 children. The limited involvement of child psychiatrists and psychologists in the treatment of autistic children by the public health system remains remarkable, having a proportion of 23.99% psychotherapies compared with the other specialized treatments such as speech therapy, occupational therapy, and special education that have a proportion of 76%. Psychotherapy and psychoeducation for people with ASD and their families are crucial for their development. In our study, children with high-functioning autism do not stand out, perhaps to avoid possible stigmatization by their social security number. Children with high-functioning autism have difficulties that are invisible to society. Education and health policies should therefore move in this direction to eliminate the stigma of diversity. Families and children with autism need support to meet their own special needs and feel like equal and productive members of a society. It is important that each country adopts the policies and takes necessary actions to ensure the development of people with autism, their well-being, and a good quality of life. A good national surveillance system for ASD could help develop understanding of the autism spectrum and monitor changes in prevalence, as well as identify possible factors contributing to the occurrence of ASD.

## Figures and Tables

**Table 1:** Nationwide prevalence of ASD by gender for children aged 2–17 years in 2021.

	Males	Females	Total
Population (N)	858,378	807,636	1,666,014
ASD (N)	12,380	3326	15,706
Prevalence	1.44%	0.41%	0.94%

**Table 2:** The prevalence of ASD in Greece in 2021 for children aged 2–17 years.

Administrative Region	Population (N)	Population ASD (N)	Prevalence of ASD (%)
Attica	585,539	6408	1.09
Eastern Macedonia–Thrace	89,046	475	0.53
North Aegean	41,635	175	0.42
Western Greece	99,241	905	0.91
Western Macedonia	38,090	270	0.71
Epirus	46,629	261	0.56
Thessaly	106,910	620	0.58
Ionian Islands	31,712	285	0.90
Central Macedonia	287,279	1594	0.55
Crete	110,257	1466	1.33
South Aegean	61,251	401	0.65
Peloponnese	84,497	765	0.91
Central Greece	83,928	657	0.78
IDs with missing locations	-	1424	
Total	1,666,014	15,706	0.94

**Table 3:** Logistic regression coefficients; the region of Attica was used as reference.

	Estimate	Odds Ratio	Std. Error	C.I.	z-Value	*p*-Value
(Intercept)	−4.50398	0.0111	0.01256	[0.0108, 0.0113]	−358.571	<0.001 ***
Eastern Macedonia–Thrace	−0.72426	0.4847	0.04769	[0.4414, 0.5322]	−15.187	<0.001 ***
North Aegean	−0.96372	0.3815	0.07679	[0.3282, 0.4434]	−12.551	<0.001 ***
Western Greece	−0.18423	0.8317	0.03568	[0.7756, 0.8920]	−5.164	<0.001 ***
Western Macedonia	−0.43819	0.6452	0.06235	[0.5710, 0.7291]	−7.028	<0.001 ***
Epirus	−0.67586	0.5087	0.06333	[0.4493, 0.5759]	−10.672	<0.001 ***
Thessaly	−0.64023	0.5272	0.04219	[0.4853, 0.5276]	−15.174	<0.001 ***
Ionian Islands	−0.19895	0.8196	0.06081	[0.7275, 0.9233]	−3.271	0.001 **
Central Macedonia	−0.68466	0.5043	0.02808	[0.4773, 0.5328]	−24.38	<0.001 ***
Crete	0.19709	1.2179	0.02914	[1.1502, 1.2894]	6.764	<0.001 ***
South Aegean	−0.51822	0.5956	0.05165	[0.5382, 0.6590]	−10.033	<0.001 ***
Peloponnese	−0.19152	0.8257	0.03843	[0.7658, 0.8903]	−4.984	<0.001 ***
Central Greece	−0.33819	0.7131	0.04113	[0.6578, 0.7729]	−8.222	<0.001 ***

** *p*-value < 0.01, *** *p*-value < 0.001.

**Table 4:** Distribution of diagnoses per sex.

Diagnosis	Sex			
	Male (N)	%	Female (N)	%
F84.0 Childhood autism	8588	67.60%	2195	63.96%
F84.1 Atypical autism	4000	31.49%	1147	33.42%
F84.2 Rett syndrome	2	0.02%	34	0.99%
F84.4 Overactive disorder associated with intellectual disability and stereotyped movements	71	0.56%	42	1.22%
F84.9 Pervasive developmental disorder, unspecified	43	0.34%	14	0.41%
Total	12,704	100%	3432	100%

**Table 5:** Proportion of diagnoses of ASD per age and sex.

Age	Sex			
	Male (N)	%	Female (N)	%
0–2	467	74.84%	157	25.16%
3–7	5888	78.27%	1635	21.73%
8–12	3989	79.97%	999	20.03%
13–17	2036	79.19%	535	20.81%
Total	12,380	78.82%	3326	21.18%

**Table 6:** Number of ASD therapies per treatment.

Treatment	N	%
Physiotherapy	2780	0.05%
Speech Therapy	1,724,618	28.19%
Occupational Therapy for Children	1,684,164	27.53%
Psychotherapy by Psychologist (Individual Sessions)	801,545	13.10%
Psychotherapy by Psychologist (Family Sessions)	6912	0.11%
Psychotherapy by Psychiatrist (Individual Sessions)	251,080	4.10%
Psychotherapy by Psychiatrist (Family Sessions)	4566	0.07%
Special Education	1,237,664	20.23%
Psychotherapy for Children by Psychologist (Group Therapy)	5698	0.09%
Psychotherapy for Children by Psychologist (Behavioral Therapy)	337,312	5.51%
Psychotherapy for Children by Psychiatrist (Group Therapy)	6582	0.11%
Psychotherapy for Children by Psychiatrist (Behavioral Therapy)	54,989	0.90%
Total	6,117,910	100.00%

**Table 7:** Annual therapies of ASD per person per age and sex.

Age	Treatments	
	Male	Female
0–2	259	270
3–7	388	378
8–12	419	422
13–17	362	341
Total	391	383

**Table 8:** Non-medication interventions for ASD per age.

Therapies	Age Group				Total
	0–2	3–7	8–12	13–17	
Physiotherapy	0.02%	0.02%	0.07%	0.07%	0.05%
Speech Therapy	30.40%	28.62%	27.77%	27.40%	28.19%
Occupational Therapy	31.14%	28.30%	27.13%	25.42%	27.53%
Psychotherapy (individual)	11.72%	14.79%	18.74%	22.14%	17.21%
Psychotherapy (family sessions)	0.37%	0.24%	0.15%	0.09%	0.19%
Special Education	13.42%	19.68%	21.25%	20.85%	20.23%
Psychotherapy (group sessions)	0.40%	0.19%	0.20%	0.22%	0.20%
Behavioral Therapy	12.53%	8.17%	4.69%	3.80%	6.41%
Total	100.00%	100.00%	100.00%	100.00%	100.00%

## Data Availability

Restrictions apply to the availability of these data. Data was obtained from EOPYY and are available from the authors with the permission of EOPYY.

## References

[1] Newschaffer C.J., Curran L.K. (2003). Autism: An emerging public health problem. Public Health Rep..

[2] Zeidan J., Fombonne E., Scorah J., Ibrahim A., Durkin M.S., Saxena S., Yusuf A., Shih A., Elsabbagh A. (2022). Global prevalence of autism: A systematic review update Search strategy. Autism Res..

[3] Tonge B.J., Dissanayake C., Brereton A.V. (1994). Autism: Fifty years on from Kanner. Paediatr. Child Health.

[4] Tuchman R. (2003). Autism. Neurol. Clin..

[5] Wolff S. (2004). The history of autism. Eur. Child Adolesc. Psychiatry.

[6] Levy F. (2016). Theories of autism. Aust. N. Z. J. Psychiatry.

[7] American Psychiatric Association (APA) (2013). Diagnostic and Statistical Manual of Mental Disorders.

[8] Faras H., Al Ateeqi N., Tidmarsh L. (2010). Autism spectrum disorders. Ann. Saudi Med..

[9] Hodges H., Fealko C., Soares N. (2020). Autism spectrum disorder: Definition, epidemiology, causes, and clinical evaluation. Transl. Pediatr..

[10] Theoharides T.C., Kavalioti M. (2019). Effect of stress on learning and motivation-relevance to autism spectrum disorder. Int. J. Immunopathol. Pharmacol..

[11] World Health Organization (2010). International Statistical Classification of Diseases and Related Health Problems.

[12] AlSalehi S.M., Alhifthy E.H. (2020). Autism spectrum disorder. Clin. Child Neurol..

[13] Schendel D., Roux A.M., Hassrick E.M., Lyall K., Shea L., Vivanti G., Wieckowski A.T., Newschaffer C., Robins D.L. (2022). Applying a public health approach to autism research: A framework for action. Autism Res..

[14] Newschaffer C.J., Croen L.A., Daniels J., Giarelli E., Grether J.K., Levy S.E., Mandell D.S., Miller L.A., Pinto-Martin J., Reaven J. (2007). The Epidemiology of Autism Spectrum Disorders. Annu. Rev. Public Health.

[15] R Core Team (2022). R: A Language and Environment for Statistical Computing.

[16] Dean M., Harwood R., Kasari C. (2017). The art of Camouflage: Gender Differences in the Social Behaviors of Girls and Boys with Autism Spectrum Disorder. Autism.

[17] Thomaidis L., Mavroeidi N., Richardson C., Choleva A., Damianos G., Bolias K., Tsolia M. (2020). Autism Spectrum Disorders in Greece: Nationwide Prevalence in 10–11 Year-Old Children and Regional Disparities. J. Clin. Med..

[18] Efthimiou A., Skounti M., Philalithis A. (2013). 2197—Prevalence and comorbidity of the autistic disorder in school age children in crete-greece. Eur. Psychiatry.

[19] Bourgeron T. (2009). A synaptic trek to autism. Curr. Opin. Neurobiol..

[20] Chiarotti F., Venerosi A. (2020). Epidemiology of autism spectrum disorders: A review of worldwide prevalence estimates since 2014. Brain Sci..

[21] Salari N., Rasoulpoor S., Rasoulpoor S., Shohaimi S., Jafarpour S., Abdoli N., Khaledi-Paveh B., Mohammadi M. (2022). The global prevalence of autism spectrum disorder: A comprehensive systematic review and meta-analysis. Ital. J. Pediatr..

[22] Will M.N., Currans K., Smith J., Weber S., Duncan A., Burton J., Kroeger-Geoppinger K., Miller V., Stone M., Mays L. (2018). Evidenced-Based Interventions for Children With Autism Spectrum Disorder. Curr. Probl. Pediatr. Adolesc. Health Care.

[23] World Health Organization. https://www.who.int/news-room/fact-sheets/detail/autism-spectrum-disorders.

